# Smoking Cessation Intention and Its Association with Advice to Quit from Significant Others and Medical Professionals

**DOI:** 10.3390/ijerph18062899

**Published:** 2021-03-12

**Authors:** Jun Hyun Hwang, Soon-Woo Park

**Affiliations:** Department of Preventive Medicine, Catholic University of Daegu School of Medicine, Daegu 42472, Korea; pmdr213@cu.ac.kr

**Keywords:** intention, advice, smoking cessation, attempt, significant others, medical professionals, Korea community health survey (KCHS)

## Abstract

Few studies have simultaneously considered the effects of significant others and medical professionals’ advice to quit smoking on smoking cessation intention. The present study involved 3841 current adult Korean smokers, divided into four groups with an intention to quit within 1 month, within 6 months, someday, and without intention to quit. Multinomial multiple logistic regression analysis was conducted according to smoking cessation intention level, adjusted for potential confounders, including past smoking cessation attempts. Smokers who had been advised to quit smoking by both significant others and medical professionals, significant others only, and medical professionals only were 2.63 (95% confidence interval (CI): 1.62–4.29), 1.84 (95% CI: 1.17–2.89), and 1.44 (95% CI: 0.70–2.94) times more likely to intend to quit within 1 month, respectively, than those who were not advised to quit. The odds ratios of an intention to quit within 6 months were 2.91 (95% CI: 1.87–4.54), 2.49 (95% CI: 1.69–3.68), and 0.94 (95% CI: 0.44–2.05), respectively. To promote smokers’ intention to quit, the role of significant others should be considered. Medical professionals’ advice to quit smoking remains important, increasing the effects of significant others’ advice.

## 1. Introduction

The use of tobacco products is among the biggest threats to human health. In 2015, worldwide, the age-standardized prevalence of daily smoking was 25.0% for men and 5.4% for women, and 11.5% of global deaths (6.4 million) were attributable to smoking [1]. This evidence suggests that programs aimed at the prevention and cessation of tobacco consumption should be among the top priorities of national public health policies.

Effective smoking cessation strategies require the understanding of the process that leads to smoking cessation and its determinants. According to the stages of change, a core construct of the transtheoretical model, smoking cessation involves a progression through precontemplation, contemplation, preparation, action, maintenance, and termination [2]. In smoking cessation behavior, these stages tend to present as three distinct states: intention to quit, an attempt to quit, and successful quitting [3]. According to the theory of planed behavior, the most important direct determinant of behavior is behavioral intention [4]. Several studies have shown that intention to quit smoking is a precursor to subsequent quitting attempts or smoking cessation. In fact, intention has been shown to account for 12% of variance in quitting success rates [5], while a strong desire to quit has been associated with a greater likelihood of quitting [6]; moreover, both those who quit and those who relapsed reported a significantly higher baseline intention to quit than persistent smokers did, and smokers’ baseline intention to quit positively predicted quitting attempts [7]. Finally, quitting attempts that lasted >1 month were significantly associated with an intention to quit in 2 months [8].

Among the various social influence factors that may affect smoking behaviors, verbal stimulation to quit smoking as a type of explicit social influence was suggested. An example of explicit verbal social influence is someone suggesting the smoker quits [9]. The most influential people who might recommend quitting tobacco use are significant others (family members, friends, and coworkers) and health professionals. An individual who attempts to change his or her health behavior may be positively influenced by their significant others during the change process [10]. For example, the proportion of explained variance in employees’ intention to quit smoking increased significantly due to social pressure from their partner and children [11]. Another study revealed that having smoking-related family conflicts was independently associated with an intention to quit either in the next 30 days or thereafter [12]. Finally, Chinese and Vietnamese adult male smokers declared that family encouragement and physician recommendation were the main facilitators in smoking cessation behavior [13].

Medical professionals’ advice to quit smoking may motivate smokers to do so and facilitate subsequent smoking cessation. A systemic review of 17 trials has shown that brief advice from physicians increased the rate of quitting 1.7-fold compared to the standard of care [14]. Smokers who received advice to quit smoking from a doctor were 1.9-fold more likely to plan to quit smoking than those who did not visit a doctor in the past 6 months [15]. In a quasi-experimental study, the advice from physicians and nurses increased the likelihood of a progression toward the action stage of the smoking cessation process, while reducing the risk of regression to the previous stage, although the latter relationship was not statistically significant [16].

Although several studies have examined the relationship between smoking cessation intentions and the advice to quit smoking from significant others or medical professionals, studies that simultaneously consider both of these factors are scarce. Such combined advice may have an additive or synergistic effect in triggering the intention to quit smoking. The aim of this study was to examine the relationship between smoking cessation advice from significant others and medical professionals and the intention to quit smoking, using the stages of behavior change as a framework.

## 2. Materials and Methods

This study used the 2017 Korea Community Health Survey (KCHS) data from Gyeongsangbuk-do province, which is among 17 regional local governments in Korea that covers 10 cities and 13 counties. The KCHS has been conducted nationwide annually since 2008 by the Korea Centers for Disease Control and Prevention to investigate community health and health-related behaviors. KCHS is conducted in 255 municipalities, where community health centers are located. Participating households are selected using stratified cluster sampling methods; all household members aged ≥ 19 years are included. Approximately 900 people per municipality participate in the survey. Qualified interviewers visit the sampled houses and collect data via face-to face interviews.

Of 22,164 participants, the present study used data of 3841 current smokers with either a daily or an occasional smoking among those who had smoked ≥ 100 cigarettes in their lifetime. The KCHS was exempted from the institutional review board review by the Korea Centers for Disease Control and Prevention. Detailed information about the KCHS is available elsewhere [17].

### 2.1. Measures

According to the transtheoretical model, the intention to quit smoking falls into four categories: (1) intention to quit within the next month (preparation stage), and (2) intention to quit within the next 6 months (contemplation stage), with the precontemplation stage subdivided into (3) intention to quit someday but not within the next 6 months, and (4) no intention to quit. There were significant differences in the characteristics of participants between those who had the intention to quit someday and those who declared having no such intention. As a result, they were included in the present study analysis, representing a separate category.

The experience of getting advice to quit smoking from others (significant others or medical professionals) was classified into four types: (1) both, (2) significant others only, (3) medical professionals only, and (4) no advice. This experience was assessed with the following questions. (1) “Do you get advice from the people around you to quit or reduce smoking?” The possible responses to this question were: “not at all”, “a little”, “somewhat”, and “always.” These responses were recoded into two categories: “yes” for “somewhat” and “always”; “no” for “not at all” and “a little”. (2) “In the past year, have you received advice from doctors, dentists, oriental doctors, or nurses to quit smoking?” The possible responses to this question were yes or no.

Other covariates were categorized into three domains: socio-demographic, smoking behavior-related, and other health-related factors. Socio-demographic variables included age (19–29, 30–39, 40–49, 50–59, 60–69, and ≥70), sex, employment (yes or no), and education level (≥college, high school, or ≤middle school). Smoking behavior-related factors included smoking frequency/amount (occasionally, ≤1/2 pack/day, ½–1 pack/day, or >1 pack/day), smoking cessation attempts (within the past year, before the past year, or never), exposure to public anti-smoking campaigns within the past year (yes or no), and experience of smoking cessation education within the past year (yes or no). Other health-related factors included frequency of alcohol drinking (≤1/month, 2–4/month, 2–3/week, ≥4/week, or missing data) and medical history of hypertension and diabetes mellitus.

### 2.2. Statistical Analysis

The distributions of covariates were calculated by four stages of intention to quit smoking, and were assessed by *χ*^2^ tests. Multinomial logistic regression analysis with the group of no intention to quit (“not at all”) used as a reference group was performed to evaluate the association between advice and intention to quit smoking. All analyses were performed using SPSS version 19.0 (IBM, Armonk, NY, USA). *p*-values of <0.05 were considered indicative of a statistically significant finding.

The stages of behavior change consider the intent to quit smoking a prerequisite for attempting to quit; however, in the case of a failure to quit, an attempt to quit may be a determinant of a continued intention to quit [18]. Accordingly, in the present study, two multivariate analyses depending on the inclusion of quit attempts were performed to analyze the association between intention to quit smoking and advice to quit from others, regardless of the impact of previous smoking cessation attempts [12].

## 3. Results

A total of 61.7% of the participants had the intention to quit smoking (within 1 or 6 months, and someday, respectively, 5.3%, 7.4%, and 49.0%). The rate of the intention to quit within 1 month was the highest among those participants who got advice from medical professionals only (7.7%), followed by medical professionals and significant others (7.0%), significant others only (4.6%), and no one (4.2%). Those who had previously attempted to quit smoking were more likely to intend to quit smoking within 1 month, compared to those who did not attempt to quit (1.2%); moreover, those who had attempted to quit smoking within the past year (13.7%) were more likely to intend to quit than those who attempted to quit more than a year before (3.9%). There were significant differences in the rates of intention to quit smoking within 1 month according to smoking frequency/amount, exposure to public anti-smoking campaigns within the past year, and experience of smoking cessation within the past year (Table 1).

Table 2 presents the results of multinomial logistic regression analyses. In the model that did not account for previous attempts at quitting, the adjusted odds ratios (ORs) for the intention to quit within 1 month (OR = 3.01, 95% confidence interval (CI): 1.89–4.79), 6 months (OR = 3.42, 95% CI: 2.22–5.27), and someday (OR = 1.69, 95% CI: 1.37–2.08) were the highest among those who got advice from both significant others and medical professionals. Advice from significant others was also significantly associated with the intention to quit smoking within 1 month (OR = 1.98, 95% CI: 1.28–3.05), 6 months (OR = 2.72, 95% CI: 1.86–3.97), and someday (OR: 1.57, 95% CI: 1.31–1.88). However, advice from medical professionals (OR = 2.14, 95% CI: 1.08–4.24) was significantly associated with the intention to quit smoking only within 1 month.

After additional adjustments for previous attempts at quitting, the adjusted ORs for the intention to quit within 1 or 6 months were the highest among the participants who got advice from both significant others and medical professionals, with OR estimates of 2.63 (95% CI: 1.62–4.29) and 2.91 (95% CI: 1.87–4.54), respectively. Of note, advice from significant others was significantly associated with the intention to quit, regardless of previous attempts at quitting. However, a significant association between advice from medical professionals and the intention to quit within 1 month was not present in the additional adjusted model including previous attempts (OR 1.44, 95% CI: 0.70–2.94).

The ORs of intention to quit within 1 month among smokers that had attempted to quit within the past year or any previous years compared to those who did not were 28.31 (95% CI: 16.10–49.77) and 4.82 (95% CI: 2.73–8.51), respectively. The ORs of the intention to quit within 6 months were 16.15 (95% CI: 10.41–25.05) in those who had attempted to quit within the past year and 4.38 (95% CI: 2.88–6.66) in those who had done so beforehand (Table 2).

## 4. Discussion

Overall, 61.7% of the study participants reported that they had an intention to quit smoking; however, only 5.3% of them intended to quit within the next month (preparation stage). This result was very low compared to results of previous studies conducted with adult smokers in Canada, where the corresponding rate was 32.5% [19], and 36% among Vietnamese male smokers in California [12]. The distribution of stages of smoking behavior in countries or groups may vary depending on the level of tobacco control policies of each group. The higher rates of intention to quit in Canada and California can be explained as a result of tobacco control policies: (1) Canada was the first country to implement pictorial warning labels in cigarette packs in 2001 and has been among the countries with the largest warning label [20]. (2) In California, the California Tobacco Control Program (CTCP), one of the longest running comprehensive tobacco control programs in the United States, has been running since 1989 [21]. In addition, the participants that intended to quit smoking within the next 6 months (contemplation stage) constituted 7.4% of the sample; overall, only 12.7% of the study participants could be considered as having the intention to quit smoking. The remaining 49.0% of the sample declared having an intention to quit smoking without a specific timeline, which is not equivalent to having an intention to quit, according to the stages of change in the transtheoretical model [2]. Nevertheless, any interpretation of smoking cessation intention rates must account for the differences in definitions between studies, for example: just want to quit smoking [3]; all of the cases, including intention to quit within the next month, the next 12 months, and will quit but not within the next 12 months [7]; separately considering the intention to quit in the next 30 days, or later but not within the next 30 days [12].

Compared to those who never attempted to quit, the ORs for the intention to quit smoking within 1 (OR: 28.3 vs. 4.8) or 6 months (OR: 16.2 vs. 4.4) were much higher for those smokers who had attempted to quit within the past year than those who attempted to quit in previous years. These findings are consistent with those of previous studies that have shown that the intention to quit smoking was stronger in the participants that had attempted to quit within the past year than that of their counterparts who had not [12,19]. In addition, even the participants that had attempted to quit more than a year before the present study were 4.8 or 4.4 times more likely to declare an intention to quit smoking within 1 or 6 months, respectively, than were those who had never tried to quit. These findings suggest that those who fail to quit may maintain a strong intention to do so. Overall, these findings suggest that smokers with a history of attempts at quitting, particularly within the past year, should be a priority group for smoking cessation programs by encouraging them to continue trying to quit smoking and providing appropriate smoking cessation interventions.

The advice to cease smoking provided by significant others was associated with the intention to quit within 1 month, 6 months, or someday, after adjusting for various potential confounders. These associations remained even after adjusting for previous attempts at quitting, which was the factor most relevant to the intention to quit, suggesting that the effect of significant others’ smoking cessation advice may be independent of other factors. Although a specific person was not defined as a “significant other” in the present study, in the Korean cultural context, these “others” are likely to include family members rather than friends or coworkers, who might regard smoking as a private matter. This presumption may be supported by the results of a prior study that social pressure from a partner and children, but not that of coworkers, influenced employees’ intentions to quit smoking [11]. The family acts as a source of social pressure and support in the social influence theory [11,22]; thus, some previous studies have emphasized the role of family in the smoking cessation process [23,24,25]. For example, family interactions related to smoking behaviors, such as the experience of smoking-related family conflicts, had a strong influence on smokers’ intentions to quit. [12]. Strategies aimed at achieving smoking cessation intention should consider not only smokers but also their families, which may play an important role in inducing smoking cessation.

Meanwhile, smoking cessation advice by medical professionals was associated with the intention to quit within 1 month in the model that did not account for previous attempts at quitting; however, this relationship did not remain significant after adjustments for previous attempts at quitting. These findings are inconsistent with those of previous studies, which have shown that the recommendation from health care professionals to quit smoking triggered smoking cessation intentions or attempts, regardless of affecting the likelihood of success [26,27,28]. The Korean government is implementing various anti-smoking policies, aimed at reducing smoking rates among men to the levels of <20%. Since 2015, these policies include economic support for smokers intending to quit, for example, varenicline prescriptions, and counseling by clinicians [29]. However, the present findings suggest that doctors’ recommendations to quit may not translate into intention to quit. Although the effect of smoking cessation advice provided by medical professionals alone was limited in the present study, it may have increased the effectiveness of such recommendations provided by significant others, in particular, among participants with the intention to quit smoking within 1 month. Regarding the intention to quit within 1 month, the advice from significant others (OR = 1.8, 95% CI: 1.2–2.9) and medical professionals (OR = 1.4, 95% CI: 0.7–2.9) showed an additive effect (OR = 2.6, 95% CI: 1.6–4.3) when provided concurrently. These findings suggest that smoking cessation policies should incorporate the advice of medical professionals and the social group of smokers.

This study has some limitations. First, this was a cross-sectional survey, which precludes any meaningful discussions about causality. Second, significant others were not defined as a specific person. Third, this study was based on a sample from a single province in Korea, limiting the generalizability of the present findings. However, covariates likely to affect the examined relationships, as previously reported, were accounted for in the present analysis [3,10,15,19]. In addition, the participants were representative of a region consisting of 23 cities or counties with a population of approximately 2.5 million, which makes this sample likely to be nationally representative. Fourth, there may be inherent limitations of the transtheoretical model related to dividing the stages arbitrarily [30]. Therefore, the results of this study should be interpreted with caution. Finally, the strength of this study is that it simultaneously considered the impact of both significant others’ and health care providers’ recommendations on smoking cessation as factors affecting smoking cessation intention.

## 5. Conclusions

Significant others, such as family members, are important to promoting smokers’ intention to quit. Although the impact of medical professionals’ advice to quit smoking may be limited, such advice is nonetheless important, as it may compound the effect of advice given by significant others, in particular, in the context of setting an intention to quit within 1 month. Moreover, smokers who have tried to quit within the previous year have a much higher willingness to quit than those who did not make such attempts. Overall, smokers require continuous encouragement and support to cease smoking.

## Figures and Tables

**Table 1:** Distribution of characteristics of participants according to the intention to quit smoking.

Classification		Intention to Quit Smoking (%)				Total (%)
		Within 1 Month	Within 6 Months	Someday	Not at All	
**Number of participants (%)**		204	285	1881	1471	3841
		5.3	7.4	49.0	38.3	100
**Main factor**						
Advised to quit smoking	Both	7.0	7.8	49.0	36.2	27.1
	Significant others	4.6	8.6	51.3	35.5	44.1
	Medical professionals	7.7	5.2	43.3	43.8	5.1
	None	4.2	5.3	45.8	44.7	23.8
		*χ*^2^ = 43.5 (*p* < 0.001)				
**Socio-demographic factor**						
Age (years)	19–29	6.6	16.7	48.7	28.0	8.3
	30–39	5.4	12.7	55.4	26.4	13.5
	40–49	5.5	8.8	55.2	30.5	20.8
	50–59	4.5	5.2	50.0	40.3	24.4
	60–69	4.7	5.3	46.0	43.9	18.7
	≥70	6.4	1.6	36.1	55.9	14.3
		χ^2^ = 220.4 (*p* < 0.001)				
Sex	Male	5.3	7.7	49.1	37.9	92.1
	Female	5.0	4.3	47.4	43.4	7.9
		χ^2^ = 6.8 (*p* = 0.080)				
Employment	Yes	5.2	7.6	50.9	36.3	79.8
	No	5.8	6.7	41.4	46.1	20.2
		*χ*^2^ = 27.7 (*p* < 0.001)				
Education level	≥College	6.7	13.2	52.2	27.8	30.1
	High school	4.6	6.7	52.5	36.2	37.9
	≤Middle school	4.8	2.8	41.7	50.8	32.0
		*χ*^2^ = 200.7 (*p* < 0.001)				
**Smoking behavior-related factor**						
Smoking frequency/amount	Occasionally	18.7	16.5	47.6	17.2	7.0
	≤1/2pack/day	5.1	8.2	52.3	34.4	34.5
	1/2~1 pack/day	3.5	6.4	48.4	41.7	50.3
	>1 pack/day	6.3	2.5	39.7	51.4	8.3
		*χ*^2^ = 211.4 (*p* < 0.001)				
Previous quit attempts	Within the past year	13.7	16	54.3	16.1	23.6
	Before the past year	3.9	6.6	56.7	32.8	42.2
	Never	1.2	2.5	35.7	60.5	34.1
		*χ*^2^ = 671.6 (*p* < 0.001)				
Exposure to public anti-smoking campaigns within the past year	Yes	5.5	8.0	49.7	36.9	90.0
	No	3.9	2.3	42.8	50.9	10.0
		*χ*^2^ = 37.5 (*p* < 0.001)				
Experience of smoking cessation education within the past year	Yes	11.6	14.7	53.9	19.8	12.1
	No	4.4	6.4	48.3	40.8	87.9
		*χ*^2^ = 126.6 (*p* < 0.001)				
**Other health-related factor**						
Hypertension	Yes	5.3	4.0	48.1	42.6	23.2
	No	5.3	8.4	49.2	37.0	76.8
		*χ*^2^ = 23.8 (*p* < 0.001)				
Diabetes mellitus	Yes	7.2	4.4	45.0	43.4	11.2
	No	5.1	7.8	49.5	37.7	88.8
		*χ*^2^ = 14.0 (*p* = 0.003)				
Frequency of alcohol drinking	≤1/month	5.7	7.9	50.7	35.7	25.5
	2~4/month	5.5	10.6	53.5	30.4	21.2
	2~3/week	4.9	8.5	50.5	36.1	24.6
	≥4/week	4.8	4.2	42.0	49	18.0
	Missing data	5.8	3.1	44.0	47.1	10.8
		*χ*^2^ = 92.5 (*p* < 0.001)				

**Table 2:** Unadjusted and adjusted odds ratios (OR, 95% confidence interval) of intention to quit smoking compared to those who had no intention.

Classification	Unadjusted OR	Model 1	Model 2
		Adjusted OR	Adjusted OR
		Excluding	Including
		Quit Attempts ^1^	Quit Attempts ^2^
**Intention to quit within 1 month**			
**Advised to quit smoking**			
Both	2.08 (1.37~3.16) ***	3.01 (1.89~4.79) ***	2.63 (1.62~4.29) ***
Significant others	1.40 (0.93~2.10)	1.98 (1.28~3.05) **	1.84 (1.17~2.89) **
Medical professionals	1.90 (1.00~3.61) *	2.14 (1.08~4.24) *	1.44 (0.70~2.94)
None	reference	reference	reference
**Smoking cessation attempts**			
Within past 1 year	42.09 (24.29~72.94) ***	-	28.31 (16.10~49.77) ***
Before past 1 year	5.87 (3.35~10.27) ***	-	4.82 (2.73~8.51) ***
Never	reference	reference	reference
**Intention to quit within 6 months**			
**Advised to quit smoking**			
Both	1.83 (1.25~2.69) **	3.42 (2.22~5.27) ***	2.91 (1.87~4.54) ***
Significant others	2.07 (1.46~2.94) ***	2.72 (1.86~3.97) ***	2.49 (1.69~3.68) ***
Medical professionals	1.00 (0.49~2.06)	1.33 (0.62~2.85)	0.94 (0.44~2.05)
None	reference	reference	reference
**Smoking cessation attempts**			
Within past 1 year	23.87 (15.73~36.22) ***	-	16.15 (10.41~25.05) ***
Before past 1 year	4.83 (3.22~ 7.25) ***	-	4.38 (2.88~6.66) ***
Never	reference	reference	reference
**Intention to quit someday**			
**Advised to quit smoking**			
Both	1.32 (1.09~1.60) **	1.69 (1.37~2.08) ***	1.49 (1.20~1.86) ***
Significant others	1.41 (1.19~1.68) ***	1.57 (1.31~1.88) ***	1.47 (1.22~1.78) ***
Medical professionals	0.97 (0.69~1.34)	1.05 (0.74~1.50)	0.80 (0.56~1.15)
None	Reference	reference	Reference
**Smoking cessation attempts**			
Within past 1 year	5.72 (4.61~7.11) ***	-	5.03 (4.01~6.32) ***
Before past 1 year	2.93 (2.51~3.43) ***	-	2.88 (2.44~3.40) ***
Never	reference	reference	reference

^1^ Adjusted for age, sex, education level, employment, smoking frequency/amount, exposure to the public campaign for smoking cessation, experience of receiving smoking cessation education, diabetes mellitus, hypertension, alcohol drinking frequency. ^2^ Additionally adjusted for quit attempts to Model 1. * *p* < 0.05, ** *p* < 0.01, *** *p* < 0.001.

## Data Availability

After approval of use, publicly available datasets were analyzed in this study. This data can be found here https://chs.cdc.go.kr/chs/rdr/rdrInfoProcessMain.do (accessed on 5 March 2021).
